# A Review of the Evidence for and against a Role for Mast Cells in Cutaneous Scarring and Fibrosis

**DOI:** 10.3390/ijms21249673

**Published:** 2020-12-18

**Authors:** Traci A. Wilgus, Sara Ud-Din, Ardeshir Bayat

**Affiliations:** 1 Department of Pathology, Ohio State University, Columbus, OH 43210, USA; 2 Centre for Dermatology Research, NIHR Manchester Biomedical Research Centre, Plastic and Reconstructive Surgery Research, University of Manchester, Manchester M13 9PT, UK; sara.ud-din@manchester.ac.uk (S.U.-D.); Ardeshir.Bayat@manchester.ac.uk (A.B.); 3 MRC-SA Wound Healing Unit, Division of Dermatology, University of Cape Town, Observatory, Cape Town 7945, South Africa

**Keywords:** mast cell, fibroblast, inflammation, hypertrophic scar, keloid, cutaneous fibrosis, scleroderma

## Abstract

Scars are generated in mature skin as a result of the normal repair process, but the replacement of normal tissue with scar tissue can lead to biomechanical and functional deficiencies in the skin as well as psychological and social issues for patients that negatively affect quality of life. Abnormal scars, such as hypertrophic scars and keloids, and cutaneous fibrosis that develops in diseases such as systemic sclerosis and graft-versus-host disease can be even more challenging for patients. There is a large body of literature suggesting that inflammation promotes the deposition of scar tissue by fibroblasts. Mast cells represent one inflammatory cell type in particular that has been implicated in skin scarring and fibrosis. Most published studies in this area support a pro-fibrotic role for mast cells in the skin, as many mast cell-derived mediators stimulate fibroblast activity and studies generally indicate higher numbers of mast cells and/or mast cell activation in scars and fibrotic skin. However, some studies in mast cell-deficient mice have suggested that these cells may not play a critical role in cutaneous scarring/fibrosis. Here, we will review the data for and against mast cells as key regulators of skin fibrosis and discuss scientific gaps in the field.

## 1. Introduction

When the skin is injured, the body initiates a wound-healing response. Depending on the tissue type and the severity of the injury, one of three general outcomes can result: regeneration of normal tissue without scarring, repair of the tissue with a scar, or fibrosis with excessive scar tissue production. Although regeneration is the ideal response to skin injury, this type of healing rarely occurs except in developing fetal skin. The normal response to tissue damage in the skin is repair. This well-characterized process begins with an inflammatory response during which resident inflammatory cells (e.g., mast cells and macrophages) become activated and circulating inflammatory cells (e.g., neutrophils and monocytes) are recruited. This is followed by a period of marked cellular proliferation and eventually the production/remodeling of collagen by fibroblasts to form a scar [1,2,3].

Scars are a significant clinical concern, and they pose many problems. Scar tissue has altered biomechanical properties, causing it to be weaker and more rigid than normal skin [4,5]. Scar tissue also inhibits the normal function of the skin, which leads to problems with thermoregulation, impairment of normal tissue growth, and restriction of joint movement. Furthermore, the quality of life for individuals with visible scars is often negatively affected [6]. In some cases, abnormal scars such as keloids or hypertrophic scars can develop. Additionally, excessive formation of scar tissue is the fundamental concern in diseases that cause cutaneous fibrosis, where there is extensive replacement of normal skin with scar tissue. Cutaneous fibrosis often develops in disorders with underlying vascular damage/dysfunction and/or pathological immune responses, such as systemic sclerosis and graft-versus-host disease.

An imbalanced or persistent inflammatory response is believed to contribute to scar formation and fibrosis [7]. One inflammatory cell type that has been proposed to drive fibroblast activation and excessive collagen deposition is the mast cell. Mast cells are resident inflammatory cells present at high numbers in organs exposed to the external environment such as the skin. Although they are known for their role in allergic reactions, mast cells can become activated in response to many different stimuli and are believed to play a role in a number physiological and pathologic processes outside of allergic responses. For example, roles for mast cells have been described in maintaining normal homeostasis, defense against parasitic, viral, and bacterial infections, neutralization/resistance to venom and toxins, and the development of diseases such as cancer and diabetes [8,9,10,11]. Mast cells are also involved in the wound repair process. Studies in animal models and human wounds have shown that mast cells undergo degranulation in response to skin injury and that mast cell numbers increase during repair, which is likely from the recruitment of mast cell precursors from the circulation [12,13,14,15,16,17]. Mast cells have been suggested to play a role in each phase of the wound repair process, including the inflammatory, proliferative, and scar formation/remodeling phases. Early after injury, they contribute to inflammatory cell recruitment and help prevent infection, they can stimulate the proliferative phase by promoting keratinocyte/fibroblast activity as well as angiogenesis, and they can communicate with fibroblasts to influence scar formation [18,19,20].

**Figure 1:**
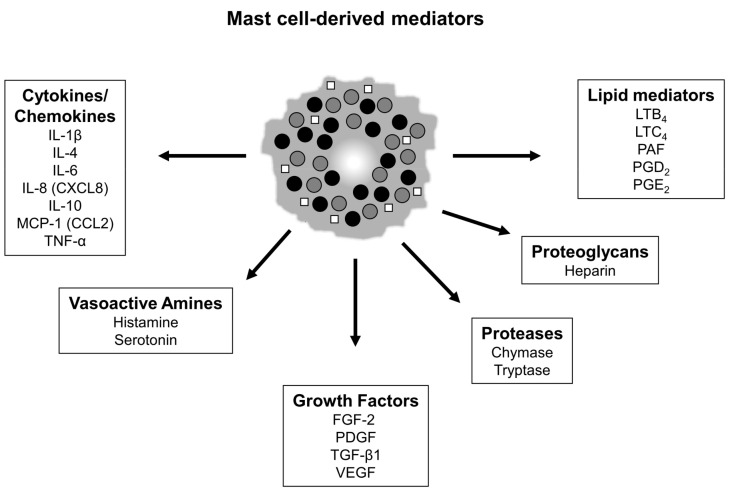
Notable mast cell mediators. Mature mast cells can secrete a wide array of mediators that are either pre-made and stored in granules or are synthesized upon activation. Mediators can be secreted from mast cells through the release of granules (filled circles) or the release of secretory vesicles (open squares). (Note: this is not a complete list of mast cell mediators).

The diversity of activities described above that have been attributed to mast cells is likely due to the ability of these cells to produce a wide range of mediators [21] (Figure 1). Many mast cell mediators can stimulate neighboring fibroblasts, which has led to a large number of studies investigating the role of mast cells in scar formation and fibrosis. The majority of published data support a pro-fibrotic role for mast cells in the skin; however, some recent studies have questioned the functional importance of these cells in fibrosis. This review will summarize the data supporting and opposing a critical role for mast cells in skin scarring and fibrosis from both human and animal studies, and will discuss open questions and future opportunities for research in this area.

## 2. Mast Cells in Scarless and Fibrotic Fetal Skin Wounds

Although most types of injury in mature skin lead to the formation of a scar, there is a period during fetal development during which regenerative, scarless healing is known to occur. Fetal skin is capable of healing scarlessly prior to the third trimester of development, whereas more mature skin beyond this stage of development heals with the formation of a fibrotic scar [22,23,24]. The extent of inflammation induced after injury is one crucial difference between scarless and fibrotic fetal wounds, and this is believed to account, at least in part, for the divergent healing outcomes. A large number of studies have shown that there is little, if any, inflammation in scarless fetal wounds; by comparison, fibrotic fetal wounds display a vigorous inflammatory response early in the healing process [25,26]. As additional evidence for the importance of inflammation in dictating the healing phenotype, the artificial induction of an inflammatory response in fetal wounds that would otherwise heal scarlessly (e.g., by introducing an inflammatory stimulus) causes the wound to heal with a scar [27,28].

Interestingly, the density of dermal mast cells is known to increase as fetal skin becomes more developed and loses its ability to heal scarlessly. This has been shown by several groups in rat [29] and mouse [30,31] skin. This also appears to be true for human skin, as it has been reported that mid-gestation fetal skin (18–22 weeks) contains a lower number of mast cells compared to mature skin (adult) [32]. In addition to an increase in mast cell density during development, mast cells become more mature with regard to their size, granular density, and the biochemical makeup of their granules [30,31,33]. These differences in mast cell maturity may explain why mast cells from less developed fetal skin do not degranulate to the same degree in response to activation stimuli in ex vivo assays or in response to injury in early-gestation scarless fetal wounds in vivo [30,34]. More detailed experiments assessing the role of mast cells in scar formation in fetal wounds showed that mast cell lysates from late-gestation fetal skin disrupted scarless healing when injected into early-gestation fetal skin wounds and that late-gestation fetal wounds created in mast cell-deficient Kit^W/W-v^ mice healed with smaller scars compared to wild-type mice that contain normal dermal mast cell numbers [30]. Together, the association between elevated mast cell numbers, enhanced mast cell maturity, and increased mast cell degranulation with the generation of scar tissue in late-gestation fetal wounds, as well as the reduction in scar formation in late-gestation wounds from mast cell-deficient mice, suggest that mast cells are an important contributor to scar formation in fetal skin (summarized in Table 1).

## 3. Mast Cells in Normal Scarring

The normal cutaneous wound repair process leads to the formation of a scar. Several studies have quantified mast cells in normal human scar tissue and found that mast cells are more abundant in scars compared to normal skin. In excisional wounds, mast cells were shown to significantly increase after injury, and the number of mast cells in 4-week and 6-week scars was approximately double the number found in uninjured skin [17]. Several other studies have reported an increase in mast cells in normal scars compared to uninjured skin [35,36,37]. Differences in the types of mast cells present in scars have also been described, with a reduction in mast cells expressing high levels of chymase and mast cells with a mature phenotype in scars, despite the higher total numbers of mast cells in scars [36,37]. This suggests that the infiltration of immature mast cell precursors may account for the increase in overall mast cell numbers in scars. Interestingly, studies have also suggested that mast cell numbers differ depending on the age of the scars, with more mast cells present in scars of more advanced age [36,37,38]. Several studies have also suggested that mast cell numbers are reduced by therapies that may mitigate scarring. For example, topical treatment with epigallocatechin-3-gallate (EGCG) was shown to improve various scar parameters, and this coincided with a reduction in mast cells in human skin wounds [17]. Overall, there appears to be a positive correlation between mast cell numbers and the normal scar formation process in human skin.

In addition to the human studies discussed above, animal models have been used to carry out functional studies testing the importance of mast cells in the normal scar formation process. These types of studies have generally used mast cell stabilizers to block mast cell degranulation or mouse strains deficient in mast cells. Disodium cromoglycate, also known as cromolyn, is a mast cell stabilizer that has been used in several animal studies. Direct injection of this drug into rat wounds has been shown to reduce collagen content [39]. In another study, systemic administration with disodium cromoglycate was shown to reduce scar size and normalize collagen architecture and collagen fibril density in murine excisional wounds [40].

Aside from mast cell stabilizers, several different mast cell-deficient mouse strains have also been used to examine the role of mast cells in normal scar formation. Kit^W/W-v^ mice are naturally occurring mutant mice that lack mast cells as a result of mutations in the gene encoding the tyrosine kinase receptor Kit. This receptor mediates signaling in response to stem cell factor, which is required for mast cell development. In a scald wound model, Kit^W/W-v^ mice were reported to heal with less wound edge fibrosis [41]. In another burn wound model, Kit^W/W-v^ mice as well as mice lacking the mast cell enzymes murine mast cell protease (mMCP)-4 (chymase) or mMCP-5 (elastase) healed with hair regrowth and less scarring [42]. In large excisional wounds, collagen remodeling was suggested to differ between wild-type and Kit^W/W-v^ mice [43]. In another excisional wound study, Kit^W/W-v^ mice were shown to produce less granulation tissue and collagen in response to negative pressure, which causes microdeformation and enhances granulation tissue formation and collagen deposition in normal wild-type mice [44].

Other studies in various mast cell-deficient strains have suggested that mast cells do not play a strong role in scar formation. In splinted excisional wounds, no differences were found in scar size or collagen density in mast cell-deficient Kit^W/W-v^, Kit^W-sh/W-sh^, or Cpa3-Cre/Mcl-1^fl/fl^ mice [45]. Kit^W/W-v^ and Kit^W-sh/W-sh^ strains have mutations in the Kit receptor, whereas Cpa3-Cre/Mcl-1^fl/fl^ mice lack mast cells due to cre recombinase-mediated ablation of the anti-apoptotic factor Mcl-1 (myeloid leukemia cell differentiation protein-1) in carboxypeptidase A (Cpa3)-expressing mast cells [46]. Another study using Cpa3^Cre/+^ mice [47], which are deficient in mast cells due to cre-related genotoxicity in mast cells, showed no differences in granulation tissue/scar area or collagen organization between mice with and without mast cells in excisional wounds [48]. Finally, a study in Mcpt5-Cre/iDTR mice [49], which allow for inducible ablation of mast cells expressing the mast cell protease 5 (Mcpt5) using diphtheria toxin administration, showed no differences in granulation tissue/scar area or alpha-smooth muscle actin (α-SMA) staining after excisional wounding [50]. The reasons behind the mixed results from mast cell-deficient mouse studies are not completely clear, but differences in wound models and time points analyzed, as well as caveats with each mouse strain could play a role (see Section 7 for more discussion on this topic). It is also possible that mast cells play a role in the scar formation process but that their functions are redundant with those of other cells, in which case the depletion of mast cells alone may not lead to obvious effects [51]. 

## 4. Mast Cells in Abnormal Scarring

Although the production of scar tissue even at normal levels can be problematic, excessive or abnormal scarring poses even more of a concern from aesthetic, functional, and clinical points of view. Hypertrophic scars (HTS) and keloids are two types of abnormal scars that can develop after injury. HTS are raised red scars that fall within the borders of the original injury, whereas keloids are abnormal raised scars that spread beyond the margins of the original wound. A number of studies have assessed the potential role of mast cells in abnormal scars.

### 4.1. Hypertrophic Scars

Several published studies have examined mast cells in human HTS. Early studies showed an increase in histamine in HTS compared to normal skin, suggesting the presence of active mast cells in HTS [52]. An increase in the incidence of allergic symptoms has also been reported in HTS patients compared to control patients [53], and burn patients with pruritis have been shown to have more mast cells and more severe scarring compared to patients without pruritis [54], suggesting a possible role for mast cell activation in scarring. Several other studies directly examining mast cells have suggested higher numbers of mast cells in HTS tissue compared to either normal skin, granulation tissue, or mature scar tissue [55,56,57,58,59,60]. Another study suggested increased staining for tryptase-positive mast cells in HTS and surgical scars compared to normal skin, although staining for chymase-positive mast cells was similar between groups [61]. In addition, several studies have suggested that various therapeutic approaches to reduce hypertrophic scar thickness, such as electric current or pressure therapy, leads to reduced dermal mast cell numbers [60,62]. In contrast to the results described above, some studies have suggested that the density of tryptase-positive mast cells is similar in HTS compared to normal surgical scars or normal skin [38,63,64,65], and one study suggested that there are reduced numbers of chymase-positive mast cells in HTS compared to normal skin [64].

Mast cells have also been examined in animal models of HTS. The red Duroc pig is often used as a model of HTS and mimics several important features of human HTS. In this model, HTS tissue was shown to have more mast cells compared to normal skin [55]. Another study in red Duroc pigs showed that treatment with the mast cell stabilizer ketotifen resulted in reduced wound contraction and collagen deposition, with the formation of thinner and less dense collagen fibrils [66]. Small animal models have also been used to study mast cells in HTS formation. Studies have shown that mast cells are prominent in HTS-like lesions that develop in mechanically loaded wounds with increased tension [67]. Elevated mast cell numbers have also been reported in a mouse model of HTS contracture [68] and in a model that used human skin grafting in nude mice to induce HTS [69].

### 4.2. Keloids

Since there is no standardized, widely accepted animal model for keloids, studies on mast cells in keloid disease have primarily been limited to descriptive studies and ex vivo evaluation of human samples. Many of the studies published to date have suggested a potential role for mast cells in keloid scarring. Elevated histamine levels [52] and high numbers of histamine-containing mast cells [70] have been described in human keloid tissue, and allergic symptoms have been reported to be more frequent in keloid-forming patients compared to the normal population [53]. The number of mast cells and degranulated (i.e., activated) mast cells have been shown to be higher in keloids compared to normal skin and normal scar tissue [35]. Other studies have suggested an increase in the number of tryptase-positive mast cells [71,72] as well as higher total mast cells, chymase-positive mast cells, and chymase enzymatic activity in keloid tissue compared to normal skin [73]. There is also some experimental evidence that reduced mast cell numbers may correlate with therapeutic effects. In one study, treatment with silicone sheeting was shown to reduce pain, pruritis, and scar height in keloids, and a reduction in mast cells was observed after treatment compared to the numbers present before treatment [74]. In addition, the green tea polyphenol EGCG has been shown to inhibit the activity of keloid fibroblasts [75,76] and reduce keloid volume in an organ culture system [77]. These effects could be due in part to the action of EGCG on mast cells, as EGCG treatment significantly reduces mast cell numbers in keloid tissue ex vivo [77]. Despite the large number of studies suggesting a positive correlation between mast cell presence and keloid scarring, some studies have reported similar numbers of mast cells in keloids compared to normal tissue [61] or low mast cell numbers in keloids [65,78,79].

Overall, the majority of studies on HTS and keloids seem to indicate that the presence of mast cells may promote abnormal scarring, but the studies reporting results contrary to this idea raise questions about whether mast cell numbers are truly associated with scar formation. Although the exact reasons for the conflicting results are not known, there are a few items that should be considered when weighing the data. First, many of the studies use different histological or immunostaining methods to identify mast cells. These methods may stain mast cell granules (toluidine blue), mast cell proteases (tryptase or chymase), or membrane receptors on mast cells (CD117/Kit), which may yield different results. Of particular importance is the fact that mast cells can degranulate and may also release proteases upon activation, making it more difficult to accurately count the number of mast cells in settings with pronounced mast cell activation in toluidine blue- or protease-stained sections. In addition, the number of samples, type of samples, and the specific area(s) within the samples being analyzed could also impact the results. For example, mast cell numbers can vary depending on the age of the scar [38,63] and can fluctuate across different regions within a scar [35]. These caveats should all be accounted for when attempting to compare or reconcile results from different studies on mast cells.

## 5. Mast Cells in Cutaneous Fibrosis

### 5.1. Systemic Sclerosis

Systemic sclerosis (SSc) is a disease characterized by fibrosis in the skin and other internal organs, and it is associated with vascular damage and immune dysfunction [80]. Many studies have shown that the number of mast cells and the frequency of mast cell degranulation is higher in sclerotic skin lesions from SSc patients compared to either uninvolved skin or skin from normal control patients, and this can depend on the type and grade of SSc [81,82,83,84,85,86,87,88]. For instance, dermal mast cells appear to be more prevalent in patients with systemic sclerosis compared to those with localized scleroderma [83], and total mast cells or degranulating mast cells have been described to be more pronounced in lower grade (scleroedematous) lesions compared to higher grade (sclerotic) lesions [83,86]. Additionally, mast cells and mast cell degranulation have also been shown to be higher in progressive lesions compared to improving or regressing lesions [81,85]. Levels of the mast cell growth factor stem cell factor (SCF) have been reported to be elevated in the skin and serum of SSc patients, which may help explain the overrepresentation of mast cells in this disease [89]. Other studies have suggested differences in the protease or cytokine profiles of mast cells in SSc lesions. For example, an increase in the frequency of MCT (tryptase^+^/chymase^−^) cells compared to MCTC (tryptase^+^/chymase^+^) cells has been described in SSc skin lesions [90], and other studies have shown that SSc is associated with an increase in the number of dermal mast cells expressing transforming growth factor (TGF)-β1/2 [88] or interleukin (IL)-17A [91].

In skin from SSc patients, there appears to be a close association between mast cells and fibroblasts, and mast cell degranulation seems to be closely tied to fibrosis [81], with degranulating mast cells prevalent in areas of severe skin fibrosis [83] and less degranulation in skin from late, regressing skin lesions [85]. Furthermore, dermal mast cell hyperactivation has been reported in a case of rapidly progressing SSc [92]. Several other studies support these observations that mast cells are in a more activated state or degranulate more readily in SSc patients. For example, plasma histamine levels were shown to be higher in SSc patients compared to healthy controls [93] suggesting higher mast cell activity, and the size of wheals generated in response to the degranulation stimulating agent 48/80 were larger in SSc patients compared to normal controls [94].

### 5.2. Mouse Models of Scleroderma

Several animal models are used to study scleroderma [95], and most studies using these models have implicated mast cells in skin fibrosis. Repeated subcutaneous injection of bleomycin is commonly used to induce skin sclerosis/fibrosis in mice. Higher numbers of total mast cells and/or numbers of degranulating mast cells have been reported in the dermis with the bleomycin model [96,97,98,99]. In experiments where bleomycin-induced skin fibrosis was alleviated due to various treatments or genetic ablation of specific mediators that reduce fibrosis, reduced mast cell density or degranulation was evident [97,100,101,102,103,104,105]. Despite the clear correlation between mast cell density and activity in bleomycin-induced fibrosis, studies in mast-cell deficient mice have yielded mixed results. One study using Kit^W/W-v^ mice showed that in early phases (1 week), sclerosis did not develop in mast cell-deficient mice, but there were no significant differences compared to control mice at later stages (2–4 weeks), suggesting that mast cells may accelerate the formation of sclerotic lesions at early stages [106]. In a more recent study using a mouse model in which diphtheria toxin was used to induce the ablation of Mcpt5-expressing mast cells, mast cell-deficient and control mice developed skin fibrosis to a similar degree in response to bleomycin at 2 and 4 weeks [50]; however, early changes were not assessed in this study.

Tight skin (Tsk) mutant mice are also commonly used as a model of skin fibrosis [95]. Fibrosis in *Tsk1*^+/^^−^ mice has been shown to be a result of a mutation in the fibrillin 1 gene [107]. An increase in mast cells and mast cell degranulation have been reported to be associated with fibrosis in *Tsk1*^+/^^−^ mice [108,109,110]. It has been suggested that the increase in mast cells in *Tsk1*^+/^^−^ mice is at least partially due to enhanced local production of cytokines such as TGF-β1, SCF, and IL-15 [109]. The mast cell stabilizers ketotifen and disodium cromoglycate, which block mast cell degranulation, were shown to inhibit fibrosis in *Tsk1*^+/^^−^ mice [111,112]. Studies have shown that the expression and activity of the mast cell protease mMCP4 (chymase) is elevated in *Tsk1*^+/^^−^ mice and treatment with a chymase inhibitor can reduce fibrosis [110,113]. In adoptive transfer experiments, the injection of bone marrow cells from *Tsk1*^+/^^−^ mice into irradiated normal mice caused the development of skin fibrosis; however, this was not associated with an increase in mast cell numbers or degranulation [114]. These results raised questions about the importance of mast cells in skin fibrosis in *Tsk1*^+/^^−^ mice. However, studies in which *Tsk1*^+/^^−^ and Kit^W/W-v^ mice were crossed to generate mast cell-deficient *Tsk1*^+/^^−^ mice suggested that while there was no change in the onset of fibrosis in the absence of mast cells, there was a reduction in the degree of fibrosis that developed in mice lacking mast cells [115].

More recent studies have suggested a role for mast cells in fibrosis using another model that has been reported to mimic several features of scleroderma. In this model, skin fibrosis develops in transgenic mice that overexpress the transcription factor Snail in epidermal keratinocytes [116]. Skin fibrosis in Snail transgenic mice is associated with an increase in dermal mast cells, which is dependent on the production of plasminogen activator inhibitor-1 (PAI-1) by keratinocytes. In vitro studies showed that PAI-1 stimulated mast cell chemotaxis and promoted mast cell-fibroblast interactions by increasing focal adhesion kinase signaling and enhancing the surface expression of intercellular adhesion molecule-1 [116].

### 5.3. Graft-Versus-Host Disease

Skin fibrosis is a recognized complication of graft-versus-host disease (GVHD), and the fibrotic process associated with GVHD shares several key features with progressive SSc [117]. An increase in mast cells has been reported in the skin of human GVHD samples compared to normal skin [118], and mast cells are commonly found in areas with irregular collagen fibers and active fibroblasts along with other immune cells [119]. Murine models of GVHD also develop cutaneous fibrosis, which is accompanied by prominent mast cell degranulation [118,120,121,122,123,124,125,126]. Studies have shown that GVHD mice treated with nedocromil sodium, a mast cell stabilizer, had less of a fibrotic response [126]. In contrast, mice treated with 48/80, which stimulates mast cell degranulation, developed more severe fibrosis [126]. In addition, less fibrosis and collagen deposition were noted in mast cell-deficient Kit^W-sh/W-sh^ mice with GVHD compared to control mice with GVHD [118].

## 6. Mechanistic Insights: Mast Cell–Fibroblast Interactions

**Figure 2:**
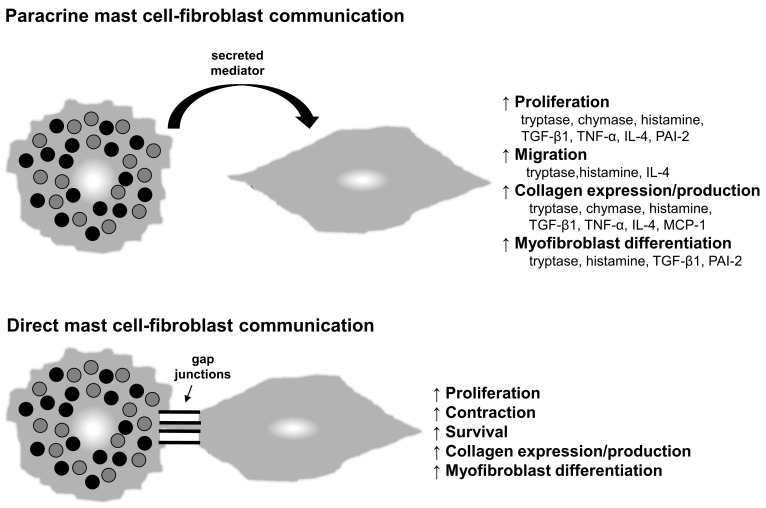
Mast cell–fibroblast interactions. Mast cells can influence the behavior of fibroblasts by secreting mediators that act in a paracrine manner (**top**) or by directly interacting with fibroblasts through heterocellular gap junctions (**bottom**).

A number of mechanisms have been described by which mast cells can stimulate the activity of dermal fibroblasts and presumably promote scar formation and fibrosis. These include the secretion of paracrine mediators by mast cells and direct mast cell–fibroblast interactions (Figure 2).

### 6.1. Paracrine Communication

Activated mast cells are known to influence the activity of fibroblasts [127,128,129,130]. Mast cells store an array of mediators in cytoplasmic granules that can be quickly released after activation through the process of degranulation. In addition, mast cells can synthesize and subsequently secrete mediators in response to activation. Many of these mast cell-derived mediators have been shown to stimulate the activity of fibroblasts, including proteases, vasoactive amines, cytokines, and growth factors (Figure 2, top panel).

Tryptase and chymase are two mast cell proteases for which a wide range of effects on dermal fibroblasts have been reported. Tryptase has been shown to enhance fibroblast proliferation [64,131,132,133], chemotaxis [134], myofibroblast differentiation (α-SMA expression) [40,135], and collagen expression or production [40,132,133,134,136]. Chymase, at least at certain concentrations, has been reported to stimulate fibroblast proliferation [64,73,137,138] and collagen expression [58,73] as well as induce TGF-β1 production and signaling [73,137,138]. Interestingly, chymase may also play a direct role in collagen biosynthesis based on in vitro studies, as cleavage of a C-terminal region of type I pro-collagen by chymase leads to de novo collagen fibril formation [139].

Histamine is a vasoactive amine produced primarily by mast cells. Although the primary target of mast cell-derived histamine may be vascular endothelial cells, histamine also affects dermal fibroblasts. Studies have shown that histamine can increase the expression of collagen [133,140] and the production of various growth factors, including TGF-β1 [140]. Histamine has also been shown to increase α-SMA expression/myofibroblast differentiation [135] as well as fibroblast proliferation [133,141,142] and migration [142].

Several other mast cell-derived mediators can affect the activity of dermal fibroblasts. Studies have shown that the ability of activated mast cells to increase collagen expression/secretion [129] and proliferation [130] are partially dependent on TGF-β1 and TNF-α (tumor necrosis factor-α). IL-4 is produced by mast cells and increases fibroblast proliferation [143], chemotaxis [144], and collagen synthesis [145,146]. Mast cell-derived PAI-2 reportedly enhances fibroblast proliferation [131] and induces α-SMA expression in fibroblasts [147], suggesting that it may stimulate myofibroblast development. Mast cells are also known to produce monocyte chemoattractant protein-1 (MCP-1/CCL2) [148,149] and several mast cell mediators, including IL-4, TGF-β1, and TNF-α, also increase the release of MCP-1 from fibroblasts [150,151]. This chemokine has been shown to increase collagen production by fibroblasts [149] and has been linked to cutaneous fibrosis/scar formation by multiple groups [152,153].

### 6.2. Direct Cell–Cell Communication

Mast cells and fibroblasts are known to be closely associated in fibrotic tissues, and studies have shown that they directly adhere to and communicate with one another via heterocellular gap junctions in vitro (Figure 2, bottom panel). The co-culture of mast cells and fibroblasts, with direct contact between the two cell types, has been shown to stimulate myofibroblast conversion and increase collagen synthesis [154], increase the rate and degree of collagen contraction in various assays [135,155,156], and increase fibroblast proliferation [143]. Direct mast cell–fibroblast interactions also appear to enhance fibroblast survival in response to dexamethasone [157]. In one study, mast cells that were degranulated prior to co-culturing were just as effective as regular mast cells in stimulating fibroblasts, suggesting that direct mast cell–fibroblast contact was responsible for the observed effects [154]. Additional studies have supported direct communication between the cells via gap junction formation by showing that connexin 43 knockdown or treatment with amide hydrolase, which both inhibit gap junction formation, blocks the ability of mast cells to directly affect fibroblast activity [154,158]. While the ability of mast cells and fibroblasts to interact directly in vitro is fairly well established, whether these interactions take place in vivo is difficult to prove. However, ultrastructural analyses of tissue have shown that mast cells and fibroblasts are in very close contact with one another in fibrotic skin and abnormal scars [81,159,160]. One published study also presented limited in vivo data suggesting that mast cells can directly communicate with fibroblasts through gap junctions in a rat polyvinyl alcohol sponge implant model by showing the transfer of dye from labeled mast cells to fibroblasts [155]. Further study in this area will be needed to fully understand the scope of mast cell–fibroblast interactions and their biological significance in vivo.

## 7. Mast Cells in Fibrosis: Unanswered Questions and Future Directions

The review of the literature presented above highlights the broad scope of published studies that have examined mast cells in cutaneous scarring and fibrosis. There is a distinct period of mast cell degranulation in response to various types of skin injury that leads to the release of mediators, many of which have been shown to stimulate dermal fibroblasts. Furthermore, based on the bulk of results from both animal studies and human samples, it seems clear that most scars and fibrotic diseases in the skin contain elevated numbers of mast cells, and these conditions are also often associated with prominent mast cell activation. Despite these key pieces of information, questions still remain about whether mast cells are actually critical for the development of scar tissue and fibrosis, primarily due to conflicting data on the functional role of dermal mast cells.

### 7.1. Experimental Limitations—Mast Cell Stabilizers

Factors that may be contributing to lingering questions about the importance of mast cells in fibrosis include limitations in the currently existing experimental approaches used to study mast cells in vivo. Mast cell stabilizers, such as ketotifen and disodium cromoglycate, have been used to prevent the release of mast cell mediators via degranulation. However, no drugs have been described that can specifically target mast cells [11] and the mast cell stabilizers described to date are somewhat non-specific and may affect other cell types, complicating interpretation of the results. In addition, the utility of these drugs for topical treatment is limited due to their chemical properties, which prevent effective penetration into the skin. New mast cell stabilizers that are more appropriate for topical use are being developed [161,162]; however, thus far, they have only been tested with the intention of treating diabetic wounds, so their usefulness for preventing scarring/fibrosis is not known.

### 7.2. Experimental Limitations—Mouse Models

In addition to mast cell stabilizers, a variety of different mast cell-deficient mouse strains have been used to study mast cell function in scar formation and cutaneous fibrosis, but the results have been variable. Many of these studies have used mice with mutations in the Kit receptor, such as Kit^W/W-v^ and Kit^W-sh/W-sh^ mice. The Kit mutations lead to mast cell deficiency, but they also cause additional abnormalities such as macrocytic anemia and neutrophil defects that can confound the results when directly comparing Kit mutant and wild-type mice [163]. One method that has been used to overcome these concerns is to compare Kit mutant mice to mutant mice that have been reconstituted with mast cells from wild-type mice via intradermal injection (sometimes referred to mast cell knock-in mice) [164]. However, thus far, all published studies on scarring and fibrosis in Kit mutant mice have directly compared wild-type and Kit mutant mice without including reconstituted/knock-in control mice. As a result, questions remain about whether any observed changes between the mouse strains in these studies are truly due to the presence or absence of mast cells. Several newer mast cell-deficient mouse strains have been developed that do not rely on Kit mutations (discussed in Section 3) [165], but these models have drawbacks as well [166]. For example, both mast cells and basophils are depleted in several of these newer strains. In addition, these models do not account for the possible existence of mast cell subpopulations with opposing functions or potential changes in these subpopulations over time, since the entire mast cell population is depleted in these strains.

### 7.3. Mast Cell Heterogeneity

**Figure 3:**
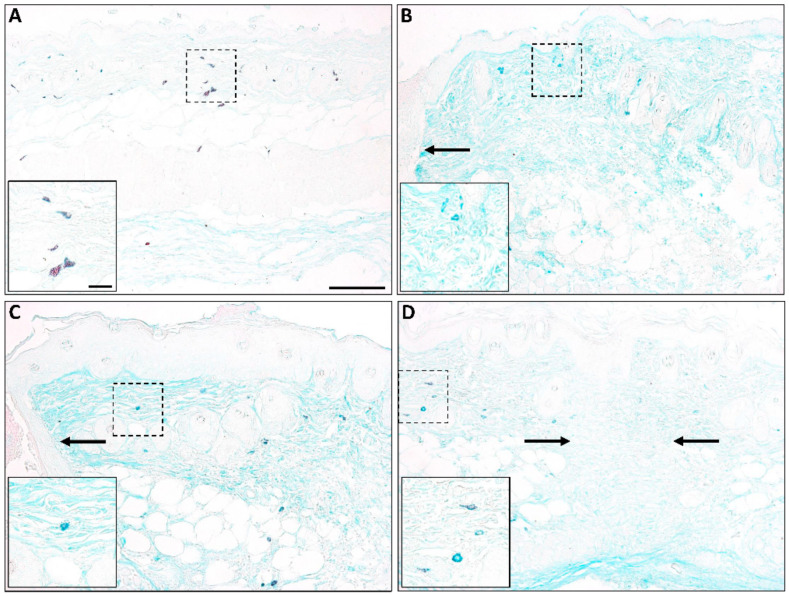
Mast cells in murine wounds. Alcian blue-safranin staining of murine skin (**A**) and wounds at 1 day (**B**), 3 days (**C**), or 7 days (**D**) post-injury shows changes in mast cell maturity during healing (blue staining = immature; red staining = mature). High numbers of mature mast cells are found in unwounded skin, followed by mostly immature mast cells at early times post-injury. By 7 days, mast cells with some red granules begin to appear. Insets show a high magnification view of the boxed area; arrows show the wound edge (**B**,**C**) and healed wound margin (**D**). Scale bar = 100 µm; Inset scale bar = 20 µm.

**Figure 4:**
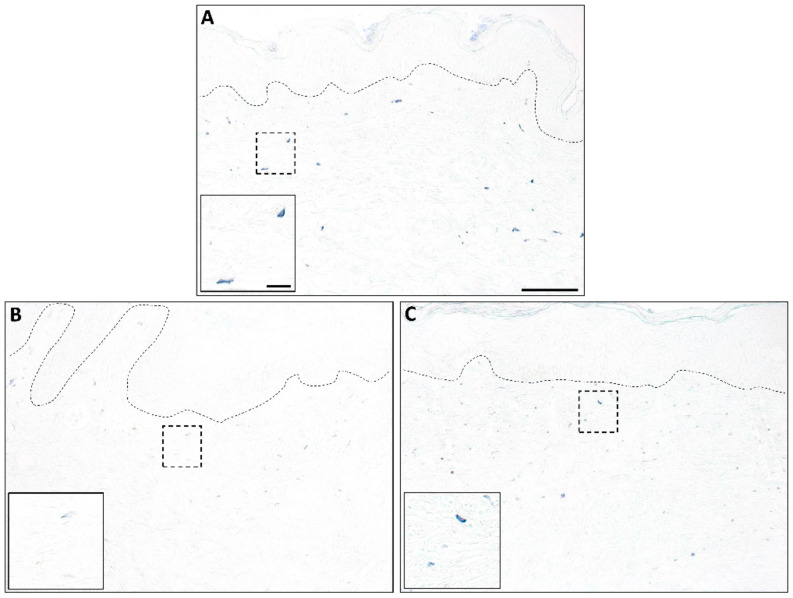
Mast cells in human wounds. Toluidine blue staining of human skin (**A**) and wounds at 4 weeks (**B**) or 8 weeks (**C**) post-injury shows changes in mast cells during healing. Mast cells with dense staining are present in unwounded skin, and an increase in mast cell numbers can be seen in 4- and 8-week wounds. Mast cells in 4-week wounds stain less intensely than those in 8-week wounds, suggesting an increase in mast cell maturity as the wounds heal. Insets show a high magnification view of the boxed area; dotted lines denote the epidermal–dermal junction. Scale bar = 100 µm; Inset scale bar = 20 µm.

The concept of mast cell heterogeneity and the possible existence of different functional mast cell subpopulations in the skin is not new, but detailed studies in this area are lacking. Published reports [36,37] and our own unpublished staining from previously collected samples [17,167] (Figure 3 and Figure 4) suggest that the characteristics of mast cells change over time in a healing wound, and it is possible that there are different mast cell subpopulations that either promote or dampen fibrosis depending on the mediators they are producing. While this idea has not been examined in detail, published studies in other experimental systems have highlighted potential mast cell heterogeneity and plasticity, as well as the possible existence of mast cell subtypes with opposing roles (e.g., mast cells with either pro-inflammatory or anti-inflammatory/immunoregulatory activity) [168,169]. The existence of a population of anti-fibrotic mast cells in some organs has been proposed [170,171,172]; however, so far, there is no evidence of this in the skin. It would be interesting to know whether there is a population of mast cells that provides stop signals late in the repair process that functions to halt the production of scar tissue, but more work needs to be done to define different mast cell subpopulations and to characterize potential changes in these cells during the scar formation process.

### 7.4. Mast Cells in Human Skin

Although there are data correlating high mast cell numbers with scarring and skin fibrosis in human skin, the relative importance of mast cells in these processes is not known. It is difficult to carry out cell-specific functional analyses in clinical studies, which is one reason animal models are commonly used to study in vivo mast cell function. Although murine models can be useful for understanding basic mechanisms of scar/fibrosis development in vivo and genetic alterations can be used to deplete mast cells or mast cell mediators, anatomical differences between mouse and human make it difficult to know how relevant results from murine studies are to human clinical disease. Further studies will have to be done to clarify the role of mast cells in cutaneous scarring and fibrosis in humans and to determine whether targeting mast cells could be a viable approach to prevent or limit scar tissue formation in human skin.

## 8. Conclusions

A comprehensive review of the published literature indicates a strong link between mast cells and cutaneous scarring/fibrosis, as well as strong evidence that mast cell-derived mediators influence fibroblast behavior. However, questions still remain about how critical mast cells are for the development of scar tissue. It is possible that mast cell activity is not absolutely required for scar formation but that mast cells promote this process. In order to better understand the role of mast cells in scar formation, more research needs to be done to characterize in detail the different mast cell subpopulations in the skin and how they change over time. More precise methods of depleting mast cells in animal models and more specific drugs to target mast cells are needed to advance the current knowledge about mast cell function in vivo. Overall, more work will be needed in order to gain a full understanding of dermal mast cells and how they contribute to the production of scar tissue during wound repair and the development of fibrosis in the skin.

## Figures and Tables

**Table 1:** Mast cell characteristics in developing skin.

		Early- to Mid-Gestation Skin (Scarless)	Late-Gestation/ Postnatal Skin (Fibrotic)
**Unwounded skin**	**Mast cell number**	lower	higher
	**Mast cell maturity**	less mature	more mature
	**Mast cell size**	smaller	larger
	**Mast cell granularity**	less dense	more dense
**Wounded skin**	**Mast cell degranulation**	infrequent	pronounced

## Abbreviations

CCL	CC chemokine ligand
Cpa3	carboxypeptidase 3
CXCL	CXC chemokine ligand
EGCG	epigallocatechin-3-gallate
FGF	fibroblast growth factor
GVHD	graft-versus-host disease
HTS	hypertrophic scar
iDTR	inducible diphtheria toxin receptor
IL	interleukin
LT	leukotriene
Mcl	myeloid leukemia cell differentiation protein
MCP-1	monocyte chemoattractant protein-1 (also known as CCL2)
Mcpt	mast cell protease
mMCP	murine mast cell protease
PAF	platelet activating factor
PAI	plasminogen activator inhibitor
PDGF	platelet derived growth factor
PG	prostaglandin
SCF	stem cell factor
SMA	smooth muscle actin
SSc	systemic sclerosis
TGF	transforming growth factor
TNF	tumor necrosis factor
Tsk	tight skin
VEGF	vascular endothelial growth factor
